# Measurement of Health-Related Quality of Life in Individuals With Rare Diseases in China: Nation-Wide Online Survey

**DOI:** 10.2196/50147

**Published:** 2023-10-31

**Authors:** Richard Huan Xu, Shamay S M Ng, Nan Luo, Dong Dong, Shuyang Zhang

**Affiliations:** 1 Department of Rehabilitation Sciences The Hong Kong Polytechnic University Kowloon China (Hong Kong); 2 Saw Swee Hock School of Public Health National University of Singapore Singapore Singapore; 3 JC School of Public Health and Primary Care The Chinese University of Hong Kong New Territories China (Hong Kong); 4 Shenzhen Research Institute The Chinese University of Hong Kong Sehnzhen China; 5 Department of Cardiology Peking Union Medical College and Chinese Academy of Medical Science Beijing China

**Keywords:** EQ-5D-5L, rare disease, normative profile, utility score, caregiver

## Abstract

**Background:**

Rare diseases (RDs) affect millions of people worldwide, and these diseases can severely impact the health-related quality of life (HRQoL) of those affected. Despite this, there is a lack of research measuring HRQoL using the EQ-5D-5L, which is one of the most widely used generic preference-based instruments to measure HRQoL in populations living with RDs.

**Objective:**

This study aimed to measure HRQoL using the EQ-5D-5L in a large number of patients with various types of RDs in China, and to examine the relationship between respondents’ socioeconomic characteristics and preference-based health utility scores.

**Methods:**

The data used in this study were obtained from a web-based survey conducted in China. The survey aimed to explore and understand the health and socioeconomic status of patients with RDs in China. We recruited registered and eligible members, including patients or their primary caregivers, from 33 RD patient associations to complete the questionnaires via their internal social networks. HRQoL was measured using the EQ-5D-5L utility score, which was calculated based on an established Chinese value set. Utility scores have been presented based on demographics and disease-related information. Univariate linear regression analysis was used to assess the differences in the EQ-5D-5L utility scores between subgroups.

**Results:**

A total of 12,502 respondents completed the questionnaire and provided valid responses, including 6919 self-completed respondents and 5583 proxy-completed respondents. Data from 10,102 participants over the age of 12 years were elicited for analysis. Among patients with RDs, 65.3% (6599/10,102), 47.5% (4799/10,102), 47.0% (4746/10,102), 24.8% (2506/10,102), and 18.4% (1855/10,102) reported no problems for “self-care,” “usual activities,” “mobility,” “pain/discomfort,” and “anxiety/depression,” respectively. A full health state was reported by 6.0% (413/6902) and 9.2% (295/3200) of self- and proxy-completed patients, respectively. Among self-completed patients, 69.9% (4826/6902) and 50.4% (3478/6902) reported no problems for “self-care” and “usual activities,” respectively, whereas only 17.7% (1223/6902) reported problems for “anxiety/depression.” Proxy-completed respondents showed a higher proportion of reporting extreme problems than self-completed respondents in all 5 dimensions. The mean utility scores reported by self- and proxy-completed respondents were 0.691 and 0.590, respectively. Different types of caregivers reported different utility scores, and among them, proxy-completed (mother) respondents reported the highest mean utility score.

**Conclusions:**

The establishment of a normative profile for RD patients can facilitate patients’ adaptation and assess the effectiveness of interventions to improve the HRQoL and well-being of this population. Differences between self- and proxy-completed HRQoL assessed by the EQ-5D-5L have been identified in this study. This finding highlights the importance of incorporating perspectives from both patients and their proxies in clinical practice. Further development of the patient cohort is necessary to assess long-term changes in HRQoL in the RD population.

## Introduction

Rare diseases (RDs) are conditions that affect a very small proportion of the population worldwide. Currently, there are approximately 7000 types of RDs, of which over 70% are genetic, and most of them show a chronic course. The prevalence threshold and definition of RDs vary across countries. In the United States and European Union, approximately 25-30 million and 45-60 million people experience RDs, respectively [1,2]. Worldwide, the population prevalence of RDs is estimated to be 3.5%-5.9%, which amounts to approximately 260-440 million people [3]. Since effective treatments for most RDs do not exist, RD patients typically require complex care, resulting in poor health status [4]. Poor access to information, high treatment expenses, and lack of social support can have a significant negative impact on their health-related quality of life (HRQoL) [5].

The EQ-5D is a well-known and widely used health status instrument worldwide. It was developed by the EuroQol Group as a concise, generic, preference-based instrument for measuring, comparing, and evaluating health status in the population aged 12 years or above [6]. It describes a person’s health as a multi-dimensional profile and provides a single utility value for it, which can be used as a HRQoL measure to facilitate the cost-utility analysis of health care and monitoring of population health [7]. The performance of the EQ-5D has been assessed in numerous health conditions and treatments, and it has shown acceptable psychometric properties. Currently, 2 versions of the EQ-5D exist: the 3-level option (3L) and the 5-level option (5L). The EQ-5D-5L is recommended owing to its better performance in reducing the “ceiling effect” compared to that of the EQ-5D-3L [8]. Currently, there is limited research on the use of the EQ-5D to measure HRQoL in the RD population as a whole. For example, Serrano-Aguilar et al used the EQ-5D for assessing HRQoL in approximately 3000 European patients and caregivers with 10 RDs [9]. Efthymiadou et al assessed HRQoL in more than 600 patients with RDs who were mostly from Europe and the United States [10], and Ng et al evaluated HRQoL in 286 patients with RDs in Hong Kong [11].

There are several gaps in the existing literature on HRQoL among individuals with RDs. First, there is a lack of research on HRQoL among Chinese patients with RDs. Currently, there are approximately 70 million Chinese patients with RDs [12], and without sufficient data and research, providing proper treatment and care for these patients is challenging. Second, although studies have recruited relatively large samples of patients with RDs in Europe, no studies with a similar sample size have been conducted in Asian countries. Random variation can have a larger impact on results in the case of small samples, potentially leading to spurious findings that might not be supported by the data. Third, no studies used the EQ-5D-5L to assess HRQoL in patients with RDs as a whole in the Chinese population. Lastly, no studies have reported the inclusion of patients with advanced-stage RDs (either subjective or objective rating). This may have generated obvious selection bias and significantly affected the quality and reliability of the findings. Therefore, the objective of this study was to measure the HRQoL of individuals or caregivers with various types of RDs using a large sample in China. Specifically, we aimed to establish a normative reference of the EQ-5D-5L for the RD population, including individuals who are older than 12 years, as a whole.

## Methods

### Sample and Data Collection

The data used in this study were obtained from a nationwide, cross-sectional, web-based survey conducted between August 2019 and January 2020. The survey aimed to explore and understand the health and socioeconomic status of patients with RDs in China. The research team comprised members from the China Alliance for Rare Disease, Chinese University of Hong Kong, China Illness Challenge Foundation, and Peking Union Medical College Hospital. In total, 32 patient associations (PAs) for RDs participated in the study. These PAs were selected from a list provided by the National Rare Diseases Registry System of China and the National Network to Collaborate on Diagnosis and Treatment of Rare Diseases. These 2 organizations were established by the Peking Union Medical College Hospital and supported by the National Health Commission of the People’s Republic of China to manage patients with RDs in China. The content of the questionnaire and logic of the survey were confirmed by the research team and representatives of all the PAs via 2 rounds of face-to-face conferences and more than 10 rounds of web-based meetings between June and August 2019.

All participants were registered members of a PA, which indicates that they were either patients with a formal diagnosis or primary caregivers of patients with a formal diagnosis. In this study, a primary caregiver was defined as a person aged ≥18 years who has been designated as a necessary caretaker responsible for managing the patient’s well-being. This may include, but is not limited to, parents, legal guardians, and paid caregivers. Since the EQ-5D-5L is not appropriate for measuring HRQoL in individuals younger than 12 years, only data from patients with RDs who were 12 years or older were elicited for analysis. Patients without cognitive impairments, who could read Chinese and provide informed consent, were encouraged to complete the questionnaire on their own. However, for all eligible participants, the option of proxy completion by caregivers was also available.

Invitations were disseminated to all eligible patients or caregivers via the PAs’ internal social networks. Eligible members who were interested in participating in the survey were invited to join an online survey group, where the study aims, process, and expected results were provided via group communication. The survey link was sent to all survey group members. The research team worked with the PA’s staff to manage the survey and remind the participants to complete it within 10 days. They sent reminders to participants on the second, third, fifth, and seventh days after the survey link was sent. The first page of the questionnaire was the informed consent form. All participants were required to read through it and click the “Agree” button at the end of the page before starting the survey. Information about participants’ background characteristics, HRQoL, symptoms, social support, and medication was collected. The research team could not access personally identifiable information of the participants; only the PAs’ staff could access such information.

### Ethics Approval

The study protocol and informed consent form were approved by the Institutional Review Board of the Chinese University of Hong Kong (Reference ID: SBRE-18-268).

### Instrument

Both the self- and proxy-completed versions of the EQ-5D-5L were used in this study [13]. The first section of the EQ-5D-5L is a descriptive system, which comprises 5 health-related dimensions (mobility, self-care, usual activities, pain/discomfort, and anxiety/depression) rated on a 5-option Likert scale ranging from no problems to extreme problems. All health states described by the descriptive system could be converted into a single utility score using a scoring algorithm based on public preferences. Utility scores are anchored between 1 (full health) and 0 (a state as bad as being dead) as required by their use in economic evaluation. A utility score of less than 0 represents health states regarded as worse than a state that is as bad as being dead. In this study, the EQ-5D-5L China value set and scoring algorithm were used [14]. The second section of the EQ-5D-5L is a visual analog scale (EQ-VAS). It is a scale that takes values between 100 (best imaginable health) and 0 (worst imaginable health), on which patients provide a global assessment of their health. The main difference between the self- and proxy-completed versions of the EQ-5D-5L is the adopted perspective. The proxy version asked respondents to report how the care receiver would rate his or her own health.

### Statistical Analysis

A descriptive analysis was applied for the participants’ demographic and health characteristics. Because self- and proxy-completed HRQoL may be systematically different, self-completed and proxy-completed EQ-5D-5L data were analyzed separately. Continuous variables (eg, EQ-5D-5L utility score) have been presented as mean, median, and SD. Categorical variables (eg, response on EQ-5D-5L dimensions) have been presented as frequency (n) and proportion (%). Pediatric patients were defined as those aged ≤18 years, whereas older patients were defined as those aged ≥60 years. Self-completed patients were defined as those who completed the questionnaire by themselves (must be ≥12 years), and proxy-completed patients (<12 years or ≥12 years but with very poor health status) were defined as those whose primary caregivers completed the questionnaire. The EQ-5D-5L profile was presented as responses on each dimension of the health classification descriptive system. The EQ-5D-5L utility score was presented for subgroups of patients stratified by their characteristics, including sex, age group, educational level, employment, family registry, family annual income, disease duration, number of children, assistive devices used in daily life, disability status, and number of family members living together, as well as the types of RDs. The univariate linear regression analysis was used to assess differences in the EQ-5D-5L utility score between subgroups. In addition, the differences in the mean EQ-5D-5L utility scores for different caregiver types (father, mother, children, spouse, grandparents/relatives, or others) stratified by background characteristics have been presented. All statistical analyses were performed using R software. Statistical significance was considered if *P*<.05 was obtained.

## Results

### Background Characteristics of Patients With RDs

A total of 20,802 responses were collected. Of these, 8300 were excluded because the respondents either dropped out of the survey or provided incomplete responses. The remaining 12,502 respondents included 6919 self-completed patients and 5583 proxy-completed patients. Table 1 shows their background characteristics stratified by response type. Of the patients, 53.2% (6653/12,502) were of the male sex, 16.2% (2016/12,502) were aged <10 years, and 3.8% (469/12,502) were aged ≥61 years, and more than half (6488/12,502, 51.9%) were urban residents. Table 1 provides a detailed breakdown of the differences. Most of the RD types were never reported previously worldwide, for example, Kallmann syndrome, Dravet syndrome, and Niemann–Pick disease. Among them, 2249 patients had myasthenia gravis, 1504 had hemophilia, and 994 had scleroderma (Multimedia Appendix 1). The self-completed and proxy-completed patients significantly differed in all the background characteristics.

**Table 1:** Patient background characteristics.

Characteristic		Full sample (N=12,502)	Self-completed sample (n=6919)	Proxy-completed sample (n=5583)	*P* value
**Gender, n (%)**					<.001
	Male	6653 (53.2)	3224 (46.6)	3429 (61.4)	
	Female	5849 (46.7)	3695 (53.4)	2154 (38.6)	
**Age (years), n (%)**					<.001
	≤10	2020 (16.2)	4 (0.1)	2016 (36.3)	
	11-20	2111 (16.9)	390 (5.6)	1721 (31.0)	
	21-30	2570 (20.6)	2128 (30.8)	442 (8.0)	
	31-40	2728 (21.9)	2367 (34.3)	361 (6.5)	
	41-50	1621 (13.0)	1319 (19.1)	302 (5.4)	
	51-60	943 (7.6)	543 (7.9)	400 (7.2)	
	≥61	469 (3.8)	146 (2.3)	313 (5.6)	
	Missing	40 (0.3)	12 (0.1)	28 (0.2)	
**Employment, n (%)**					<.001
	Active	3578 (28.6)	3019 (43.6)	559 (10.0)	
	Nonactive	8924 (71.4)	3900 (56.4)	5024 (90.0)	
**Family registry, n (%)**					<.001
	Urban	6488 (51.9)	3783 (54.7)	2705 (48.5)	
	Rural	5991 (48.0)	3120 (45.1)	2871 (51.4)	
	Missing	23 (0.1)	7 (0.2)	16 (0.3)	
**Family income per year (CNY^a^), n (%)**					<.001
	≤5000	705 (5.6)	433 (6.3)	272 (4.9)	
	5001-10,000	782 (6.3)	427 (6.2)	355 (6.4)	
	10,001-30,000	2584 (20.7)	1367 (19.8)	1217 (21.8)	
	30,001-50,000	2923 (23.4)	1459 (21.1)	1464 (26.2)	
	50,001-100,000	3228 (25.8)	1823 (26.3)	1405 (25.2)	
	100,001-200,000	1554 (12.4)	955 (13.8)	599 (10.7)	
	200,001-300,000	417 (3.3)	263 (3.8)	154 (2.8)	
	300,001-500,000	196 (1.6)	125 (1.8)	71 (1.3)	
	≥500,001	113 (0.9)	67 (1.0)	46 (0.8)	
**Duration of RDs^b^ (years), n (%)**					<.001
	≤10	4709 (37.6)	2681 (38.7)	2028 (36.3)	
	11-20	4909 (39.2)	2214 (32.0)	2695 (48.3)	
	21-30	1545 (12.3)	1145 (16.5)	400 (7.2)	
	31-40	696 (5.5)	593 (8.6)	1032 (18.5)	
	≥41	302 (2.4)	260 (3.8)	42 (0.8)	
	Missing	341 (2.7)	26 (0.4)	315 (5.6)	
**Number of children, n (%)**					<.001
	0	7230 (57.8)	2968 (42.9)	4262 (76.3)	
	1	3516 (28.1)	2816 (40.7)	700 (12.5)	
	2	1438 (11.5)	989 (14.3)	449 (8.0)	
	≥3	318 (2.5)	146 (2.1)	172 (3.1)	
**Using assistive devices in daily life (eg, wheelchair, ventilator, etc), n (%)**					<.001
	No	7200 (57.6)	4074 (58.9)	3126 (58.9)	
	Rarely	1887 (15.1)	1134 (16.4)	753 (16.4)	
	Sometimes	1490 (11.9)	852 (12.3)	639 (11.4)	
	Often	950 (7.6)	458 (6.6)	492 (8.8)	
	Always	958 (7.7)	396 (5.7)	562 (10.1)	
**Disability (either physical or psychological), n (%)**					<.001
	Yes	3551 (28.4)	1880 (27.2)	1671 (29.9)	
	No	8951 (71.6)	5039 (72.8)	3912 (71.1)	
**Number of family members living together, n (%)**					<.001
	0	480 (3.8)	271 (3.9)	209 (3.7)	
	1	1855 (14.8)	1140 (16.5)	715 (12.8)	
	2	3457 (27.6)	1975 (28.5)	1482 (26.5)	
	3	2725 (21.7)	1304 (18.8)	1421 (25.5)	
	≥4	3155 (25.2)	1585 (22.9)	1570 (28.1)	
	Missing	830 (6.6)	644 (9.3)	186 (3.3)	
Perceived disease severity (score 1-10), mean (SD)		8.2 (2.3)	7.8 (2.3)	8.7 (2.1)	<.001

^a^A currency exchange rate of CNY 1=US $0.14 is applicable.

^b^RD: rare disease.

### EQ-5D-5L Profile for Patients With RDs

Data from 10,102 participants over the age of 12 years were elicited for analysis. Their background characteristics are presented in Multimedia Appendix 2. Table 2 displays the proportion of reported health states for each level of the EQ-5D-5L dimensions. Among patients with RDs, 65.3% (6599/10,102), 47.5% (4799/10,102), 47.0% (4746/10,102), 24.8% (2506/10,102), and 18.4% (1855/10,102) reported no problems for “self-care,” “usual activities,” “mobility,” “pain/discomfort,” and “anxiety/depression,” respectively. In the case of pediatric patients with RDs, 49.3% (704/1428), 40.2% (574/1428), and 28.6% (408/1428) reported no problems for “usual activities,” “pain/discomfort,” and “anxiety/depression,” respectively, and these proportions were around 10% higher than the proportions in the overall sample. The proportions of having no problems in all 5 dimensions were lower in older patients with RDs than in the overall sample.

Among self-completed respondents, 69.9% (4826/6902) and 50.4% (3478/6902) reported no problems for “self-care” and “usual activities,” respectively, whereas only 17.7% (1223/6902) reported problems for “anxiety/depression.” Moreover, 6.0% (413/6902) and 0.3% (24/6902) of patients reported full and worst health states, respectively.

**Table 2:** Health status reported by using the EQ-5D-5L descriptive system.

Variable		All patients (N=10,102)	Pediatric (12-18 years) (n=1428)	Older (≥60 years) (n=503)	Self-completed (n=6902)	Proxy-completed (n=3200)
**Mobility, n (%)**						
	No problem	4746 (47.0)	802 (56.2)	134 (26.6)	3311 (48.0)	1435 (44.8)
	Slight problems	2495 (24.7)	219 (15.3)	135 (26.8)	1844 (26.7)	651 (20.3)
	Moderate problems	1311 (13.0)	125 (8.8)	84 (16.7)	917 (13.3)	394 (12.3)
	Severe problems	744 (7.4)	63 (4.4)	70 (13.9)	477 (6.9)	267 (8.3)
	Extreme problems	806 (8.0)	219 (15.3)	80 (15.9)	353 (5.1)	453 (14.2)
**Self-care, n (%)**						
	No problem	6599 (65.3)	872 (61.1)	212 (42.1)	4826 (69.9)	1773 (55.4)
	Slight problems	1751 (17.3)	190 (13.3)	113 (22.5)	1221 (17.7)	530 (16.6)
	Moderate problems	746 (7.4)	98 (6.9)	67 (13.3)	466 (6.8)	280 (8.8)
	Severe problems	345 (3.4)	45 (3.2)	39 (7.8)	190 (2.8)	155 (4.8)
	Extreme problems	661 (6.5)	223 (15.6)	72 (14.3)	199 (2.9)	462 (14.4)
**Usual activities, n (%)**						
	No problem	4799 (47.5)	704 (49.3)	127 (25.2)	3478 (50.4)	1321 (41.3)
	Slight problems	2862 (28.3)	322 (22.5)	162 (32.2)	2028 (29.4)	834 (26.1)
	Moderate problems	1222 (12.1)	140 (9.8)	89 (17.7)	826 (12.0)	396 (12.4)
	Severe problems	638 (6.3)	83 (5.8)	66 (13.1)	374 (5.4)	264 (8.3)
	Extreme problems	581 (5.8)	179 (12.5)	59 (11.7)	196 (2.8)	385 (12.0)
**Pain/discomfort, n (%)**						
	No problem	2506 (24.8)	574 (40.2)	77 (15.3)	1606 (23.3)	900 (28.1)
	Slight problems	4540 (44.9)	533 (37.3)	198 (39.4)	3292 (47.7)	1248 (39.0)
	Moderate problems	2055 (20.3)	196 (13.7)	132 (26.2)	1424 (20.6)	631 (19.7)
	Severe problems	647 (6.4)	72 (5.0)	62 (12.3)	399 (5.8)	248 (7.8)
	Extreme problems	354 (3.5)	53 (3.7)	34 (6.8)	181 (2.6)	173 (5.4)
**Anxiety/depression, n (%)**						
	No problem	1855 (18.4)	408 (28.6)	72 (14.3)	1223 (17.7)	632 (19.8)
	Slight problems	4407 (43.6)	656 (45.9)	178 (35.4)	3066 (44.4)	1341 (41.9)
	Moderate problems	2379 (23.5)	220 (15.4)	140 (27.8)	1695 (24.6)	684 (21.4)
	Severe problems	888 (8.8)	80 (5.6)	64 (12.7)	571 (8.3)	317 (9.9)
	Extreme problems	573 (5.7)	64 (4.5)	49 (9.7)	347 (5.0)	226 (7.1)
Full health^a^, n (%)		708 (7.0)	214 (15.0)	21 (4.2)	413 (6.0)	295 (9.2)
Worst health^b^, n (%)		74 (0.7)	13 (0.9)	17 (3.4)	24 (0.3)	50 (1.6)

^a^Full health indicates that the respondent selected “no problem” for all 5 dimensions of the EQ-5D descriptive system (1, 1, 1, 1, and 1).

^b^Worst health indicates that the respondent selected “severe/extreme problems” for all 5 dimensions of the EQ-5D descriptive system (5, 5, 5, 5, and 5).

Proxy-completed respondents showed a higher proportion of reporting extreme problems than self-completed respondents in all 5 dimensions. Among proxy-completed respondents, 55.4% (1773/3200) and 44.8% (1435/3200) reported no problems for “self-care” and “mobility,” respectively. Regarding the types of caregivers, fathers reported the highest proportion of care receivers experiencing problems related to extreme mobility and self-care (91/535, 17.0% and 84/535, 15.7%, respectively), whereas children caregivers reported a higher proportion of care receivers experiencing extreme problems with usual activities and pain/discomfort (69/595, 11.6% and 47/595, 7.9%) than other types of caregivers (Table 3). Meanwhile, grandparents/relatives and children caregivers reported similarly high proportions of extreme problems with anxiety/depression (approximately 11%) compared to other types of caregivers.

**Table 3:** Proxy-completed health status using the EQ-5D-5L descriptive system.

Variable			Father (n=535)		Mother (n=1084)		Children (n=595)		Spouse (n=369)		Grandparents/relatives (n=158)		Others (n=459)
**Mobility, n (%)**													
	No problem	258 (48.2)		570 (52.6)		208 (35.0)		143 (38.8)		56 (35.4)		200 (43.6)	
	Slight problems	99 (18.5)		188 (17.3)		137 (23.0)		93 (25.2)		35 (22.2)		99 (21.6)	
	Moderate problems	56 (10.5)		91 (8.4)		100 (16.8)		50 (13.6)		30 (19.0)		67 (14.6)	
	Severe problems	31 (5.8)		64 (5.9)		76 (12.8)		39 (10.6)		16 (10.1)		41 (8.9)	
	Extreme problems	91 (17.0)		171 (15.8)		74 (12.4)		44 (11.9)		21 (13.3)		52 (11.3)	
**Self-care, n (%)**													
	No problem	293 (54.8)		634 (58.5)		283 (47.6)		205 (55.6)		77 (48.7)		281 (61.2)	
	Slight problems	88 (16.4)		152 (14.0)		110 (18.5)		78 (21.1)		33 (20.9)		69 (15.0)	
	Moderate problems	43 (8.0)		80 (7.4)		71 (11.9)		23 (6.2)		20 (12.7)		43 (9.4)	
	Severe problems	27 (5.0)		43 (4.0)		41 (6.9)		19 (5.1)		10 (6.3)		15 (3.3)	
	Extreme problems	84 (15.7)		175 (16.1)		90 (15.1)		44 (11.9)		18 (11.4)		51 (11.1)	
**Usual activities, n (%)**													
	No problem	225 (42.1)		500 (46.1)		199 (33.4)		143 (38.8)		48 (30.4)		206 (44.9)	
	Slight problems	132 (24.7)		257 (23.7)		172 (28.9)		107 (29.0)		48 (30.4)		118 (25.7)	
	Moderate problems	64 (12.0)		105 (9.7)		89 (15.0)		50 (13.6)		29 (18.4)		59 (12.9)	
	Severe problems	38 (7.1)		75 (6.9)		66 (11.1)		28 (7.6)		17 (10.8)		40 (8.7)	
	Extreme problems	76 (14.2)		147 (13.6)		69 (11.6)		41 (11.1)		16 (10.1)		36 (7.8)	
**Pain/discomfort, n (%)**													
	No problem	175 (32.7)		391 (36.1)		121 (20.3)		82 (22.2)		25 (15.8)		106 (23.1)	
	Slight problems	212 (39.6)		424 (39.1)		211 (35.5)		166 (45.0)		65 (41.1)		170 (37.0)	
	Moderate problems	85 (15.9)		163 (15.0)		153 (25.7)		70 (19.0)		45 (28.5)		115 (25.1)	
	Severe problems	30 (5.6)		66 (6.1)		63 (10.6)		32 (8.7)		12 (7.6)		45 (9.8)	
	Extreme problems	33 (6.2)		40 (3.7)		47 (7.9)		19 (5.1)		11 (7.0)		23 (5.0)	
**Anxiety/depression, n (%)**													
	No problem	127 (23.7)		272 (25.1)		79 (13.3)		59 (16.0)		16 (10.1)		79 (17.2)	
	Slight problems	234 (43.7)		485 (44.7)		219 (36.8)		167 (45.3)		63 (39.9)		173 (37.7)	
	Moderate problems	98 (18.3)		196 (18.1)		144 (24.2)		82 (22.2)		47 (29.7)		117 (25.5)	
	Severe problems	35 (6.5)		86 (7.9)		88 (14.8)		38 (10.3)		15 (9.5)		55 (12.0)	
	Extreme problems	41 (7.7)		45 (4.2)		65 (10.9)		23 (6.2)		17 (10.8)		35 (7.6)	
Full health^a^, n (%)			55 (10.3)		136 (12.5)		36 (6.1)		20 (5.4)		5 (3.2)		43 (9.4)
Worst health^b^, n (%)			9 (1.7)		7 (0.6)		17 (2.9)		6 (1.6)		4 (2.5)		7 (1.5)

^a^Full health indicates that the respondent selected “no problem” for all 5 dimensions of the EQ-5D descriptive system (1, 1, 1, 1, and 1).

^b^Worst health indicates that the respondent selected “severe/extreme problems” for all 5 dimensions of the EQ-5D descriptive system (5, 5, 5, 5, and 5).

### EQ-5D-5L Utility Score for Patients With RDs

Tables 4-6 show the means and SDs of the EQ-5D-5L utility scores stratified by different background characteristics and types of RDs. The mean utility scores were 0.659 (SD 0.324), 0.691 (SD 0.284), and 0.590 (SD 0.388) for the overall, self-completed, and proxy-completed samples, respectively. In the overall sample, the EQ-5D-5L utility score was significantly associated with belonging to the female sex, being aged ≤10 years, being actively employed, residing in urban areas, having a high family annual income, having a short duration of disease, and not having children. Similar trends were observed in the self- and proxy-completed samples, except for insignificant differences in the utility score between patients with different numbers of children in the self-completed sample and between urban and rural residents in the proxy-completed sample. Regarding the mean utility score for specific RDs, patients with amyotrophic lateral sclerosis reported a significantly lower utility score than those with other RDs; however, in the self-completed sample, the mean utility score for spinal muscular atrophy was the lowest (Multimedia Appendix 3).

Regarding the EQ-5D-5L utility scores reported by different types of caregivers, we found that mothers reported a higher score (0.633) whereas children caregivers reported a lower score (0.513) compared to other caregivers (0.552). Detailed comparisons of the utility scores of caregivers are provided in Table 7.

**Table 4:** EQ-5D utility scores and the associations with patients’ background characteristics (full sample).

Variable		Utility score, mean (SD)	Coefficient (95% CI)	*P* value
Overall		0.659 (0.324)	N/A^a^	N/A
**Gender**				
	Male	0.633 (0.339)	N/A	N/A
	Female	0.685 (0.308)	0.051 (0.039 to 0.064)	<.001
**Age (years)**				
	12-20	0.660 (0.362)	N/A	N/A
	21-30	0.709 (0.289)	0.049 (0.030 to 0.068)	<.001
	31-40	0.698 (0.278)	0.038 (0.019 to 0.057)	<.001
	41-50	0.625 (0.319)	−0.035 (−0.057 to 0.014)	.001
	51-60	0.557 (0.364)	−0.104 (−0.129 to 0.079)	<.001
	≥61	0.476 (0.412)	−0.185 (−0.217 to 0.152)	<.001
**Employment**				
	Active	0.787 (0.206)	N/A	N/A
	Nonactive	0.589 (0.355)	−0.198 (−0.211 to −0.186)	<.001
**Family registry**				
	Urban	0.669 (0.322)	N/A	N/A
	Rural	0.647 (0.327)	−0.021 (−0.034 to −0.008)	.001
**Family annual income (CNY^b^)**				
	≤5000	0.505 (0.381)	N/A	N/A
	5001-10,000	0.543 (0.367)	0.038 (0.003 to 0.074)	.03
	10,001-30,000	0.602 (0.334)	0.098 (0.069 to 0.127)	<.001
	30,001-50,000	0.658 (0.316)	0.153 (0.125 to 0.182)	<.001
	50,001-100,000	0.702 (0.299)	0.197 (0.169 to 0.225)	<.001
	100,001-200,000	0.746 (0.277)	0.241 (0.211 to 0.272)	<.001
	200,001-300,000	0.751 (0.287)	0.246 (0.204 to 0.288)	<.001
	300,001-500,000	0.755 (0.283)	0.250 (0.195 to 0.305)	<.001
	≥500,001	0.753 (0.315)	0.249 (0.180 to 0.317)	<.001
**Duration of RDs^c^ (years)**				
	≤10	0.710 (0.291)	N/A	N/A
	11-20	0.644 (0.340)	−0.066 (−0.081 to −0.052)	<.001
	21-30	0.617 (0.340)	−0.093 (−0.112 to −0.074)	<.001
	31-40	0.591 (0.317)	−0.119 (−0.145 to −0.093)	<.001
	≥41	0.499 (0.348)	−0.211 (−0.249 to −0.174)	<.001
**Number of children**				
	0	0.669 (0.329)	N/A	N/A
	1	0.664 (0.311)	−0.005 (−0.019 to 0.009)	.48
	2	0.641 (0.323)	−0.028 (−0.047 to −0.009)	.005
	≥3	0.530 (0.369)	−0.139 (−0.175 to −0.102)	<.001
**Using assistive devices in daily life (eg, wheelchair, ventilator, etc)**				
	No	0.822 (0.177)	N/A	N/A
	Rarely	0.616 (0.242)	−0.206 (−0.219 to −0.193)	<.001
	Sometimes	0.518 (0.285)	−0.304 (−0.318 to −0.290)	<.001
	Often	0.316 (0.338)	−0.506 (−0.524 to −0.488)	<.001
	Always	0.089 (0.336)	−0.733 (−0.751 to −0.716)	<.001
**Disability (either physical or psychological)**				
	Yes	0.450 (0.377)	N/A	N/A
	No	0.742 (0.258)	0.293 (0.280 to 0.306)	<.001
**Number of family members living together**				
	0	0.584 (0.372)	N/A	N/A
	1	0.629 (0.343)	0.045 (0.011 to 0.078)	.009
	2	0.674 (0.318)	0.089 (0.057 to 0.122)	<.001
	3	0.662 (0.322)	0.078 (0.045 to 0.111)	<.001
	≥4	0.662 (0.316)	0.077 (0.045 to 0.110)	<.001

^a^N/A: not applicable.

^b^A currency exchange rate of CNY 1=US $0.14 is applicable.

^c^RD: rare disease.

**Table 5:** EQ-5D utility scores of the self-completed sample.

Variable		Utility score, mean (SD)	Coefficient (95% CI)	*P* value
Overall		0.691 (0.284)	N/A^a^	N/A
**Gender**				
	Male	0.664 (0.298)	N/A	N/A
	Female	0.715 (0.270)	0.051 (0.037 to 0.064)	<.001
**Age (years)**				
	12-20	0.709 (0.294)	N/A	N/A
	21-30	0.719 (0.274)	0.009 (−0.021 to 0.040)	.53
	31-40	0.708 (0.266)	−0.002 (−0.032 to 0.028)	.91
	41-50	0.649 (0.298)	−0.061 (−0.092 to −0.029)	<.001
	51-60	0.631 (0.308)	−0.078 (−0.115 to −0.042)	<.001
	≥61	0.584 (0.358)	−0.126 (−0.178 to −0.073)	<.001
**Employment**				
	Active	0.793 (0.195)	N/A	N/A
	Nonactive	0.612 (0.316)	−0.181 (−0.194 to −0.168)	<.001
**Family registry**				
	Urban	0.700 (0.281)	N/A	N/A
	Rural	0.680 (0.288)	−0.020 (−0.034 to −0.007)	.003
**Family income per year (CNY^b^)**				
	≤5000	0.530 (0.353)	N/A	N/A
	5001-10,000	0.577 (0.330)	0.047 (0.010 to 0.084)	.01
	10,001-30,000	0.627 (0.300)	0.097 (0.068 to 0.127)	<.001
	30,001-50,000	0.680 (0.275)	0.150 (0.120 to 0.179)	<.001
	50,001-100,000	0.738 (0.251)	0.208 (0.179 to 0.237)	<.001
	100,001-200,000	0.786 (0.220)	0.256 (0.225 to 0.287)	<.001
	200,001-300,000	0.790 (0.230)	0.260 (0.218 to 0.302)	<.001
	300,001-500,000	0.806 (0.223)	0.276 (0.221 to 0.331)	<.001
	≥500,001	0.793 (0.276)	0.263 (0.192 to 0.333)	<.001
**Duration of RDs^c^ (years)**				
	≤10	0.750 (0.238)	N/A	N/A
	11-20	0.693 (0.286)	−0.057 (−0.072 to −0.410)	<.001
	21-30	0.640 (0.311)	−0.109 (−0.129 to −0.090)	<.001
	31-40	0.602 (0.310)	−0.148 (−0.172 to −0.123)	<.001
	≥41	0.498 (0.347)	−0.251 (−0.287 to −0.216)	<.001
**Number of children**				
	0	0.686 (0.298)	N/A	N/A
	1	0.697 (0.276)	0.011 (−0.004 to 0.026)	.14
	2	0.694 (0.269)	0.008 (−0.013 to 0.028)	.45
	≥3	0.670 (0.265)	−0.016 (−0.063 to 0.032)	.52
**Using assistive devices in daily life (eg, wheelchair, ventilator, etc)**				
	No	0.824 (0.159)	N/A	N/A
	Rarely	0.634 (0.222)	−0.190 (−0.204 to −0.176)	<.001
	Sometimes	0.533 (0.269)	−0.291 (−0.307 to −0.275)	<.001
	Often	0.381 (0.327)	−0.443 (−0.464 to −0.423)	<.001
	Always	0.187 (0.348)	−0.638 (−0.660 to −0.615)	<.001
**Disability (either physical or psychological)**				
	Yes	0.510 (0.347)	N/A	N/A
	No	0.759 (0.222)	0.249 (0.235 to 0.263)	<.001
**Number of family members living together**				
	0	0.683 (0.277)	N/A	N/A
	1	0.672 (0.307)	−0.010 (−0.048 to 0.027)	.58
	2	0.689 (0.284)	0.006 (−0.030 to 0.043)	.73
	3	0.692 (0.281)	0.009 (−0.028 to 0.046)	.63
	≥4	0.699 (0.275)	0.016 (−0.021 to 0.053)	.40

^a^N/A: not applicable.

^b^A currency exchange rate of CNY 1=US $0.14 is applicable.

^c^RD: rare disease.

**Table 6:** EQ-5D utility scores of the proxy-completed sample.

Variable		Utility score, mean (SD)	Coefficient (95% CI)	*P* value
Overall		0.590 (0.388)	N/A^a^	N/A
**Gender**				
	Male	0.579 (0.394)	N/A	N/A
	Female	0.603 (0.381)	0.024 (−0.003 to 0.051)	.09
**Age (years)**				
	12-20	0.646 (0.378)	N/A	N/A
	21-30	0.661 (0.345)	0.014 (−0.026 to 0.055)	.49
	31-40	0.637 (0.337)	−0.010 (−0.053 to 0.034)	.67
	41-50	0.521 (0.381)	−0.125 (−0.173 to −0.078)	<.001
	51-60	0.456 (0.408)	−0.191 (−0.233 to −0.149)	<.001
	≥61	0.422 (0.427)	−0.225 (−0.271 to −0.178)	<.001
**Employment**				
	Active	0.755 (0.255)	N/A	N/A
	Nonactive	0.556 (0.402)	−0.199 (−0.234 to −0.164)	<.001
**Family registry**				
	Urban	0.599 (0.390)	N/A	N/A
	Rural	0.578 (0.388)	−0.02 (−0.047 to 0.007)	.14
**Family income per year (CNY^b^)**				
	≤5000	0.442 (0.439)	N/A	N/A
	5001-10,000	0.475 (0.425)	0.034 (0.384 to 0.499)	.39
	10,001-30,000	0.551 (0.391)	0.109 (−0.043 to 0.111)	.001
	30,001-50,000	0.616 (0.378)	0.175 (0.045 to 0.174)	<.001
	50,001-100,000	0.625 (0.370)	0.183 (0.111 to 0.238)	<.001
	100,001-200,000	0.642 (0.368)	0.200 (0.121 to 0.246)	<.001
	200,001-300,000	0.638 (0.387)	0.196 (0.130 to 0.270)	<.001
	300,001-500,000	0.591 (0.381)	0.150 (0.099 to 0.294)	.03
	≥500,001	0.655 (0.384)	0.214 (0.016 to 0.283)	.007
**Duration of RDs^c^ (years)**				
	≤10	0.617 (0.372)	N/A	N/A
	11-20	0.569 (0.398)	−0.066 (−0.095 to −0.036)	<.001
	21-30	0.552 (0.405)	−0.083 (−0.126 to −0.039)	<.001
	31-40	0.530 (0.348)	−0.104 (−0.182 to −0.027)	.009
	≥41	0.504 (0.359)	−0.130 (−0.249 to −0.011)	.03
**Number of children**				
	0	0.643 (0.372)	N/A	N/A
	1	0.531 (1.400)	−0.111 (−0.144 to −0.078)	<.001
	2	0.526 (2.393)	−0.116 (−0.156 to −0.077)	<.001
	≥3	0.411 (0.402)	−0.232 (−0.292 to −0.172)	<.001
**Using assistive devices in daily life (eg, wheelchair, ventilator, etc)**				
	No	0.817 (0.215)	N/A	N/A
	Rarely	0.573 (0.279)	−0.244 (−0.270 to −0.217)	<.001
	Sometimes	0.485 (0.315)	−0.332 (−0.360 to −0.303)	<.001
	Often	0.207 (0.329)	−0.610 (−0.643 to −0.576)	<.001
	Always	−0.015 (0.289)	−0.832 (−0.861 to −0.803)	<.001
**Disability (either physical or psychological)**				
	Yes	0.336 (0.403)	N/A	N/A
	No	0.705 (0.322)	0.369 (0.343 to 0.395)	<.001
**Number of family members living together**				
	0	0.439 (0.442)	N/A	N/A
	1	0.540 (0.393)	0.102 (0.037 to 0.166)	.002
	2	0.639 (0.382)	0.201 (0.139 to 0.262)	<.001
	3	0.606 (0.382)	0.167 (0.104 to 0.230)	<.001
	≥4	0.580 (0.378)	0.141 (0.079 to 0.204)	<.001

^a^N/A: not applicable.

^b^A currency exchange rate of CNY 1=US $0.14 is applicable.

^c^RD: rare disease.

**Table 7:** EQ-5D-5L utility scores stratified by the types of caregivers.

Variable			Utility score, mean (SD)												
			Father		Mother		Children		Spouse		Grandparents/relatives		Others		
Overall			0.603 (0.396)		0.633 (0.377)		0.513 (0.409)		0.588 (0.382)		0.530 (0.375)		0.595 (0.371)		
**Gender**															
	Male	0.559 (0.411)		0.618 (0.383)		0.552 (0.401)		0.573 (0.409)		0.541 (0.362)		0.564 (0.383)			
	Female	0.732 (0.316)		0.653 (0.369)		0.483 (0.414)		0.602 (0.356)		0.515 (0.392)		0.645 (0.345)			
**Age (years)**															
	12-20	0.628 (0.390)		0.653 (0.376)		N/A^a^		N/A		0.631 (0.354)		0.548 (0.416)			
	21-30	0.588 (0.415)		0.666 (0.347)		N/A		0.791 (0.186)		0.601 (0.302)		0.669 (0.337)			
	31-40	0.695 (0.320)		0.536 (0.441)		N/A		0.677 (0.331)		0.584 (0.361)		0.630 (0.320)			
	41-50	0.562 (0.306)		0.480 (0.314)		0.583 (0.339)		0.498 (0.416)		0.435 (0.383)		0.529 (0.413)			
	51-60	N/A		0.468 (0.408)		0.460 (0.406)		0.458 (0.408)		0.234 (0.395)		0.443 (0.402)			
	≥61	N/A		N/A		0.405 (0.437)		0.393 (0.467)		N/A		0.443 (0.466)			
**Employment**															
	Active	0.755 (0.285)		0.762 (0.245)		0.685 (0.290)		0.775 (0.230)		0.722 (0.237)		0.782 (0.252)			
	Nonactive	0.589 (0.402)		0.623 (0.384)		0.483 (0.420)		0.470 (0.412)		0.463 (0.391)		0.502 (0.385)			
**Family registry**															
	Urban	0.634 (0.388)		0.643 (0.371)		0.515 (0.424)		0.574 (0.396)		0.489 (0.375)		0.603 (0.364)			
	Rural	0.566 (0.405)		0.617 (0.387)		0.509 (0.394)		0.608 (0.362)		0.559 (0.373)		0.588 (0.377)			
**Family income per year (CNY^b^)**															
	≤5000	0.391 (0.451)		0.547 (0.412)		0.329 (0.469)		0.396 (0.488)		0.449 (0.395)		0.446 (0.427)			
	5001-10,000	0.492 (0.450)		0.502 (0.437)		0.493 (0.420)		0.395 (0.419)		0.357 (0.414)		0.495 (0.415)			
	10,001-30,000	0.573 (0.431)		0.579 (0.384)		0.491 (0.400)		0.484 (0.410)		0.510 (0.362)		0.571 (0.347)			
	30,001-50,000	0.606 (0.386)		0.661 (0.360)		0.524 (0.427)		0.670 (0.325)		0.636 (0.331)		0.605 (0.369)			
	50,001-100,000	0.627 (0.370)		0.670 (0.364)		0.571 (0.370)		0.604 (0.373)		0.504 (0.414)		0.643 (0.353)			
	100,001-200,000	0.709 (0.350)		0.693 (0.350)		0.481 (0.416)		0.617 (0.356)		0.588 (0.328)		0.728 (0.301)			
	200,001-300,000	0.787 (0.244)		0.594 (0.412)		0.553 (0.407)		0.763 (0.377)		N/A		0.679 (0.365)			
	300,001-500,000	0.494 (0.346)		0.609 (0.434)		0.450 (0.430)		0.803 (0.143)		N/A		0.710 (0.410)			
	≥500,001	0.775 (0.169)		0.660 (0.326)		0.330 (0.488)		0.877 (0.111)		N/A		0.928 (0.144)			
**Duration of RDs^c^ (years)**															
	≤10	0.620 (0.386)		0.663 (0.362)		0.536 (0.397)		0.629 (0.367)		0.599 (0.299)		0.675 (0.336)			
	11-20	0.595 (0.391)		0.602 (0.390)		0.508 (0.414)		0.522 (0.419)		0.444 (0.390)		0.553 (0.396)			
	21-30	0.544 (0.464)		0.633 (0.347)		0.383 (0.442)		0.553 (0.349)		0.542 (0.467)		0.570 (0.391)			
	31-40	0.318 (0.364)		0.501 (0.374)		0.453 (0.447)		0.592 (0.276)		0.717 (0.093)		0.571 (0.325)			
	≥41	N/A		0.625 (0.330)		0.644 (0.275)		0.385 (0.544)		0.436 (0.422)		0.395 (0.357)			
**Number of children**															
	0	0.624 (0.393)		0.651 (0.373)		0.699 (0.341)		0.757 (0.218)		0.601 (0.337)		0.608 (0.367)			
	1	0.511 (0.426)		0.544 (0.384)		0.469 (0.412)		0.557 (0.396)		0.457 (0.410)		0.596 (0.375)			
	2	0.572 (0.365)		0.494 (0.372)		0.476 (0.417)		0.608 (0.383)		0.493 (0.397)		0.588 (0.350)			
	≥3	0.403 (0.396)		0.417 (0.418)		0.386 (0.400)		0.622 (0.355)		0.310 (0.341)		0.364 (0.463)			
**Using assistive devices in daily life (eg, wheelchair, ventilator, etc)**															
	No	0.838 (0.218)		0.835 (0.210)		0.786 (0.222)		0.804 (0.193)		0.756 (0.259)		0.806 (0.215)			
	Rarely	0.636 (0.240)		0.588 (0.297)		0.501 (0.298)		0.605 (0.236)		0.527 (0.238)		0.586 (0.280)			
	Sometimes	0.440 (0.354)		0.498 (0.322)		0.431 (0.342)		0.485 (0.293)		0.484 (0.270)		0.552 (0.264)			
	Often	0.202 (0.321)		0.244 (0.337)		0.173 (0.317)		0.159 (0.318)		0.070 (0.274)		0.269 (0.361)			
	Always	0.007 (0.288)		0.059 (0.283)		−0.115 (0.245)		−0.133 (0.246)		−0.030 (0.255)		0.009 (0.343)			
**Disability (either physical or psychological)**															
	Yes	0.336 (0.400)		0.381 (0.393)		0.237 (0.405)		0.246 (0.419)		0.343 (0.409)		0.350 (0.403)			
	No	0.732 (0.324)		0.777 (0.280)		0.597 (0.372)		0.679 (0.315)		0.607 (0.332)		0.732 (0.268)			
**Number of family members living together**															
	0	0.563 (0.402)		0.533 (0.400)		0.333 (0.442)		0.491 (0.490)		0.423 (0.340)		0.401 (0.470)			
	1	0.507 (0.462)		0.603 (0.381)		0.471 (0.400)		0.585 (0.352)		0.405 (0.429)		0.594 (0.360)			
	2	0.663 (0.403)		0.672 (0.370)		0.607 (0.410)		0.549 (0.389)		0.531 (0.331)		0.652 (0.357)			
	3	0.641 (0.360)		0.616 (0.382)		0.601 (0.383)		0.557 (0.419)		0.566 (0.402)		0.577 (0.378)			
	≥4	0.530 (0.391)		0.607 (0.383)		0.505 (0.384)		0.676 (0.332)		0.679 (0.284)		0.561 (0.373)			

^a^N/A: not applicable.

^b^A currency exchange rate of CNY 1=US $0.14 is applicable.

^c^RD: rare disease.

### EQ-VAS Profile

**Figure 1:**
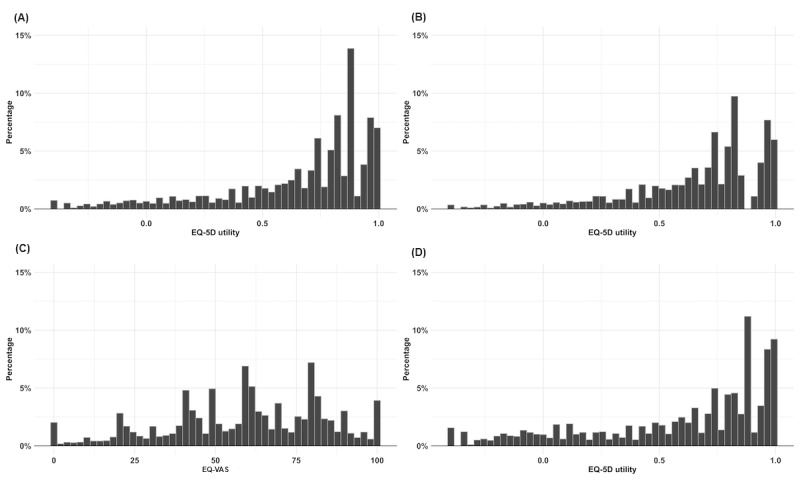
Distribution of the EQ-5D-5L utility scores and EQ-VAS scores. (A) EQ-5D utility score for the full sample; (B) EQ-5D utility score for the self-completed sample; (C) EQ-VAS score for the full sample; (D) EQ-5D utility score for the proxy-completed sample.

The mean and median EQ-VAS scores of the full sample were 59.22 and 61, respectively, with scores ranging from 0 to 100. The mean and median EQ-VAS scores of the self- and proxy-completed samples were 60.19 and 61, and 57.13 and 60, respectively. There was a strong and significant association between the EQ-5D-5L utility score and the EQ-VAS score (r=0.52; *P*<.001). Additionally, Figure 1 presents the distribution of the EQ-VAS and EQ-5D-5L utility scores.

## Discussion

### Principal Findings

This study presents a population norm of HRQoL for patients with RDs in China, using the preference-based value set of the EQ-5D-5L. This is also the largest study estimating the utility scores of the EQ-5D-5L in patients with RDs worldwide, even including studies using the EQ-5D-3L or any other preference-based measures. Although we only included 33 types of RDs, it is still the largest number of RDs included in any EQ-5D-5L–related study worldwide. Additionally, we estimated the EQ-5D-5L profile and utility scores for both pediatric and older patients with RDs, as well as self-completed and proxy-completed patients with RDs, and evaluated the differences between caregiver types. Our study provides valuable information as a reference for health evaluation and comparisons of different health care interventions in RDs, both locally and globally.

### Comparisons With Previous Studies

The mean utility score for patients with RDs (0.659) in our sample, as measured by the EQ-5D-5L, was significantly lower than that for the general Chinese population in China (0.957) [15] and Hong Kong (0.92) [16]. This score is comparable to the findings of López-Bastida et al [17], who utilized a sample of 1544 patients with RDs from several European countries. Our mean utility score is similar to theirs, suggesting that our results are consistent with those of other large-scale studies conducted in the RD population. However, López-Bastida et al did not report the respondents’ selections on the dimensions of the EQ-5D. This makes it difficult to compare dimension-level data between the 2 studies. Moreover, given that the value set for China and European countries may be different, the index score between this study and the Europeans study may not be comparable. Compared with another study using the EQ-5D-3L in Chinese patients with RDs in Hong Kong [11], there were higher proportions of “no problems” for the dimensions of “mobility,” “self-care,” and “usual activities,” but lower proportions of “no problems” for “pain/discomfort” and “anxiety/depression.” Additionally, regarding the utility score, the Hong Kong study reported a lower mean score (0.53) than our study. However, the Hong Kong study had a very small sample size, which may have limited the reliability of their findings. Compared to studies assessing HRQoL in Chinese patients with chronic diseases in general [18-22], the utility scores in patients with RDs were significantly lower. It reflects the large negative impact of RDs on HRQoL and the greater health care needs of patients with RDs in China.

Similar to the general population norm [15,16,23-25], demographics and socioeconomics also significantly impacted patients with RDs. For example, older patients with RDs living in rural areas or having lower family annual incomes reported significantly lower utility scores than their counterparts. Regarding disease-related factors, we found a very large difference (0.733) between patients with RDs who did not use assistive devices and those who used them in daily life. Despite the global need and recognized benefits of using assistive products, access to them remains limited [26]. Our findings quantify the importance of assistive devices for improving the HRQoL of patients with RDs and promoting their well-being. Moreover, we have further presented the EQ-5D-5L utility score for all 33 types of RDs. We found that patients with neuromuscular diseases (amyotrophic lateral sclerosis and spinal muscular disease) tended to report lower utility scores compared to the scores in patients with other types of RDs. This is in line with previous findings. For example, Ng et al [11] and Sequeira et al [27] indicated that patients with neurologic diseases reported the lowest utility scores among patients of all RD categories. Despite acknowledging that some studies have measured HRQoL based on disease category (eg, neurologic disease) rather than a single disease, we decided not to categorize the 33 RDs into different categories, because even though some RDs share similar pathologic and physiologic mechanisms, each RD has its own characteristics. Our list can help researchers in flexibly comparing HRQoL in their research based on either a single RD or a category of RDs.

The EQ-5D-5L proxy questionnaire has been shown to be feasible and valid in various populations. However, most studies have focused on people with mental health problems, such as dementia [28,29]. The EQ-5D-5L profile in patients with RDs is limited. Given that a high proportion of RD patients are children or adolescents, owing to the genetic nature of RDs, proxy-completed data are very useful in RD research. Our study provides valuable information about EQ-5D-5L utility scores completed by proxies. We found that the mean utility score of proxy-completed patients was lower than that of self-completed patients, which is consistent with previous findings [30]. This is reasonable as most RD patients who cannot complete the questionnaire themselves have poor physical and mental health. However, for this study, we only used the proxy-patient version of the EQ-5D-5L, and HRQoL measured by the proxy-proxy version may be assessed in the future.

In addition, to provide a more detailed analysis, we have further stratified the EQ-5D utility score based on the type of caregiver. Our findings are similar to those of a previous study reporting that different proxies provide different ratings of patient health [31]. Mothers reported a higher proportion of “no problems” for nearly all dimensions of the EQ-5D-5L than fathers and grandparents or relatives. However, fathers reported a higher proportion of “extreme problems” for 4 out of 5 dimensions than mothers. This may be because when the mother is the primary caregiver, the health condition of the child is usually not very severe, allowing the father to go out to work. However, if the father is the primary caregiver, both parents may have caregiving responsibilities and the child’s health condition is often much more serious. Additionally, most child caregivers reported a lower proportion of “no problems” than other types of caregivers, as the care receivers of these caregivers are usually older patients with RDs. These data are useful because, compared to patients with normal disease, more adult patients with RDs cannot complete the EQ-5D by themselves owing to their poor health status. Measuring HRQoL by caregivers is therefore important to examine the effectiveness of interventions that are designed to improve the practice of RD care.

### Strengths and Limitations

This study presents a comprehensive analysis of a normative profile of the EQ-5D-5L for patients with RDs in China. We used a large sample size and included both self- and proxy-completed data to provide a more complete picture of HRQoL for this population than that provided by previous studies worldwide. Our findings can serve as a baseline for future comparisons of HRQoL with RD populations, both locally and globally. Moreover, the results can be used to evaluate the efficacies of various policies, strategies, or interventions aimed at improving the HRQoL of the RD population. Furthermore, the study provides EQ-5D-5L utility values for the future cost-effectiveness analysis of interventions for RDs. By providing a detailed and nuanced understanding of the EQ-5D-5L normative profile for patients with RDs, this study can help inform future research and policy initiatives designed to address the unique health needs of this population.

Despite these strengths, several limitations should be addressed. The primary limitation of this study is that all data were collected voluntarily, which may have led to a lack of information from patients with a poor health status or caregivers with low willingness to participate in the survey, generating selection bias. Additionally, all data were collected via a web-based survey, which could exclude patients not familiar with or not having easy access to the internet, potentially leading to selection bias. Moreover, in this study, we used the EQ-5D-5L instead of the EQ-5D-Y to collect HRQoL data from children and adolescents with RDs. Given that the EQ-5D-Y is a youth-friendly version that has demonstrated high feasibility and reliability in young patients, the estimation of the utility score in this population in our sample might be less reliable. In the future, it is highly encouraged to conduct studies that use EQ-5D-Y to assess HRQoL in young patients with RDs. Finally, although the sample size in our study is larger than that in previous studies, it is important to note that there may be over 20 million people living with RDs in China. Therefore, our sample may not be fully representative, and the generalizability of our findings could be limited.

### Conclusion

This study assessed the HRQoL of Chinese patients with RDs using the preference-based EQ-5D-5L. Our findings contribute new knowledge to the existing literature on the relationship among HRQoL, demographics, and health status in patients with RDs. The established normative profile of HRQoL reveals disparities and heterogeneities existing in the health status, as measured by the EQ-5D-5L, across different socioeconomic groups and disease categories. Additionally, our study revealed important differences between self- and proxy-completed HRQoL assessments in the RD population. This finding underscores the need to incorporate perspectives from both patients and their proxies in clinical practice. Further development of the patient cohort is necessary to assess the long-term changes in HRQoL in the RD population.

## Appendix

Multimedia Appendix 1 Patient characteristics stratified by the types of rare diseases.

Multimedia Appendix 2 Background characteristics of patients aged 12 years or older.

Multimedia Appendix 3 EQ-5D-5L utility scores reported by specific rare diseases.

## Abbreviations

HRQoL: health-related quality of life
PA: patient association
RD: rare disease
VAS: visual analog scale

## Data Availability

Derived data supporting the findings of this study are available from the corresponding author on request.
