# Supplementary material for: Measurement of Health-Related Quality of Life in Individuals With Rare Diseases in China: Nation-Wide Online Survey
Source: JMIR Public Health Surveill. 2023 Oct 31;9:e50147. doi: 10.2196/50147 (PMC10646671; doi:10.2196/50147)
Supplement: Multimedia Appendix 1 [file publichealth_v9i1e50147_app1.docx]

**Multimedia Appendix 1.** Patient characteristics stratified by the types of rare diseases.

| **Types of RDs, n (%)** | | Full sample (N=12,502) | Self-completed (N=6919) | Proxy-completed (N=5583) | *P* value |
| --- | --- | --- | --- | --- | --- |
|  | Myasthenia gravis | 2249 (18.0) | 1751 | 498 | <.001 |
|  | Hemophilia | 1504 (12.0) | 916 | 588 |  |
|  | Scleroderma | 944 (7.5) | 686 | 258 |  |
|  | Marfan syndrome | 929 (7.4) | 575 | 354 |  |
|  | Phenylketonuria | 781 (6.3) | 37 | 744 |  |
|  | Multiple sclerosis | 739 (5.9) | 619 | 120 |  |
|  | Duchenne muscular dystrophy | 701 (5.6) | 15 | 686 |  |
|  | Tuberous sclerosis complex | 436 (3.5) | 106 | 330 |  |
|  | Hepatolenticular degeneration | 399 (3.2) | 257 | 142 |  |
|  | Spinal muscular atrophy | 338 (2.7) | 90 | 248 |  |
|  | Neuromyelitis optica spectrum disorders | 331 (2.6) | 264 | 67 |  |
|  | Epidermolysis bullosa | 317 (2.5) | 170 | 147 |  |
|  | Huntington’s disease | 266 (2.1) | 35 | 231 |  |
|  | Lymphangioleiomyomatosis | 257 (2.1) | 250 | 7 |  |
|  | Congenital adrenal hyperplasia | 228 (1.8) | 16 | 212 |  |
|  | Spinal and bulbar muscular atrophy | 214 (1.7) | 159 | 55 |  |
|  | Albinism | 197 (1.6) | 118 | 79 |  |
|  | Amyotrophic lateral sclerosis | 196 (1.6) | 68 | 128 |  |
|  | Fabry disease | 189 (1.5) | 140 | 49 |  |
|  | Spinocerebellar ataxia | 181 (1.4) | 135 | 46 |  |
|  | Mucopolysaccharidosis type I | 153 (1.2) | 13 | 140 |  |
|  | Kallmann syndrome | 141 (1.1) | 128 | 13 |  |
|  | Osteogenesis imperfecta | 135 (1.1) | 84 | 51 |  |
|  | Pompe disease | 117 (0.9) | 83 | 34 |  |
|  | Prader-Willi syndrome | 114 (0.9) | 1 | 113 |  |
|  | Gaucher disease | 100 (0.8) | 39 | 61 |  |
|  | Idiopathic hypogonadotropic hypogonadism | 88 (0.7) | 83 | 5 |  |
|  | Langerhans cell histiocytosis | 66 (0.5) | 42 | 24 |  |
|  | Tetrahydrobiopterin deficiency | 58 (0.5) | 4 | 54 |  |
|  | Dravet syndrome | 49 (0.4) | 0 | 49 |  |
|  | Niemann-Pick disease | 33 (0.3) | 1 | 32 |  |
|  | Idiopathic pulmonary artery hypertension | 31 (0.2) | 24 | 7 |  |
|  | Homozygote familial hypercholesterolemia | 21 (0.2) | 10 | 11 |  |
