# Supplementary material for: Measurement of Health-Related Quality of Life in Individuals With Rare Diseases in China: Nation-Wide Online Survey
Source: JMIR Public Health Surveill. 2023 Oct 31;9:e50147. doi: 10.2196/50147 (PMC10646671; doi:10.2196/50147)
Supplement: Multimedia Appendix 2 [file publichealth_v9i1e50147_app2.docx]

**Multimedia Appendix 2.** Background characteristics of patients aged 12 years or older.

|  | Full sample | | Self-completed | Proxy-completed |  |
| --- | --- | --- | --- | --- | --- |
|  | N=10,102 | % | N=6902 | N=3200 | p-value |
| Gender |  |  |  |  |  |
| Male | 5051 | 50.0 | 3215 | 1836 | <.001 |
| Female | 5051 | 50.0 | 3687 | 1364 |  |
| Age |  |  |  |  |  |
| 12-20 | 1771 | 17.5 | 389 | 1382 | <.001 |
| 21-30 | 2570 | 25.4 | 2128 | 442 |  |
| 31-40 | 2728 | 27.0 | 2367 | 361 |  |
| 41-50 | 1621 | 16.0 | 1319 | 302 |  |
| 51-60 | 943 | 9.3 | 543 | 400 |  |
| >=61 | 469 | 4.6 | 156 | 313 |  |
| Employment |  |  |  |  |  |
| Active | 3561 | 35.3 | 3015 | 546 | <.001 |
| Non-active | 6541 | 64.7 | 3887 | 2654 |  |
| Family registry |  |  |  |  |  |
| Urban | 5490 | 54.3 | 3772 | 1718 | <.001 |
| Rural | 4590 | 45.4 | 3115 | 1475 |  |
| Missing | 22 | 0.2 | 15 | 7 |  |
| Family income per year (CNY) |  |  |  |  |  |
| <5000 | 603 | 6.0 | 430 | 173 | <.001 |
| 5001 ~10,000 | 643 | 6.4 | 427 | 216 |  |
| 10,001~30,000 | 2028 | 20.1 | 1364 | 664 |  |
| 30,001~50,000 | 2226 | 22.0 | 1454 | 772 |  |
| 50,001~100,000 | 2671 | 26.4 | 1818 | 853 |  |
| 100,001~200,000 | 1319 | 13.1 | 955 | 364 |  |
| 200,001~300,000 | 355 | 3.5 | 263 | 92 |  |
| 300,001~500,000 | 163 | 1.6 | 124 | 39 |  |
| >500,001 | 94 | 0.9 | 67 | 27 |  |
| Duration of RDs (years) |  |  |  |  |  |
| ≤ 10 | 3808 | 37.7 | 2670 | 1138 | <.001 |
| 11~20 | 3653 | 36.2 | 2213 | 1440 |  |
| 21~30 | 1544 | 15.3 | 1144 | 400 |  |
| 31~40 | 695 | 6.9 | 592 | 103 |  |
| ≥ 41 | 302 | 3.0 | 260 | 42 |  |
| Missing | 100 | 1.0 | 23 | 77 |  |
| Number of children |  |  |  |  |  |
| 0 | 4842 | 47.9 | 2958 | 1884 | <.001 |
| 1 | 3508 | 34.7 | 2811 | 697 |  |
| 2 | 1435 | 14.2 | 987 | 448 |  |
| ≥ 3 | 317 | 3.1 | 146 | 171 |  |
| Using assistive devices in daily life (e.g., wheelchair or ventilator, etc.) |  |  |  |  |  |
| No | 5733 | 56.8 | 4063 | 1670 | <.001 |
| Rarely | 1619 | 16.0 | 1131 | 488 |  |
| Sometimes | 1236 | 12.2 | 850 | 386 |  |
| Often | 732 | 7.2 | 458 | 274 |  |
| Always | 765 | 7.6 | 395 | 370 |  |
| Disability (either physical or psychological) |  |  |  |  |  |
| Yes | 2874 | 28.4 | 1876 | 998 | <.001 |
| No | 7228 | 71.6 | 5026 | 2202 |  |
| Number of family member living together |  |  |  |  |  |
| 0 | 453 | 4.5 | 270 | 183 | <.001 |
| 1 | 1698 | 16.8 | 1140 | 558 |  |
| 2 | 2854 | 28.3 | 1967 | 887 |  |
| 3 | 2002 | 19.8 | 1301 | 701 |  |
| >=4 | 2300 | 22.8 | 1580 | 720 |  |
| Missing | 795 | 7.9 | 644 | 151 |  |
| Type of RDs |  |  |  |  |  |
| Myasthenia gravis | 2191 | 21.7 | 1749 | 442 | <.001 |
| Hemophilia | 1280 | 12.7 | 916 | 364 |  |
| Scleroderma | 886 | 8.8 | 684 | 202 |  |
| Marfan syndrome | 877 | 8.7 | 571 | 306 |  |
| Phenylketonuria | 271 | 2.7 | 36 | 235 |  |
| Multiple sclerosis | 734 | 7.3 | 617 | 117 |  |
| Duchenne muscular dystrophy | 228 | 2.3 | 15 | 213 |  |
| Tuberous sclerosis complex | 256 | 2.5 | 106 | 150 |  |
| Hepatolenticular degeneration | 374 | 3.7 | 257 | 117 |  |
| Spinal Muscular atrophy | 187 | 1.9 | 90 | 97 |  |
| Neuromyelitis optica spectrum disorders | 324 | 3.2 | 261 | 63 |  |
| Epidermolysis bullosa | 229 | 2.3 | 170 | 59 |  |
| Huntington's disease | 264 | 2.6 | 35 | 229 |  |
| Lymphangioleiomyomatosis | 256 | 2.5 | 249 | 7 |  |
| Congenital adrenal hyperplasia | 69 | 0.7 | 16 | 53 |  |
| Spinal and bulbar muscular atrophy | 214 | 2.1 | 159 | 55 |  |
| Albinism | 139 | 1.4 | 118 | 21 |  |
| Amyotrophic lateral sclerosis | 196 | 1.9 | 68 | 128 |  |
| Fabry disease | 183 | 1.8 | 139 | 44 |  |
| Spinocerebellar ataxia | 181 | 1.8 | 135 | 46 |  |
| Mucopolysarcharidosis type I | 66 | 0.7 | 13 | 53 |  |
| Kallmann syndrome | 141 | 1.4 | 128 | 13 |  |
| Osteogenesis imperfecta | 112 | 1.1 | 83 | 29 |  |
| Pompe disease | 111 | 1.1 | 83 | 28 |  |
| Prader-Willi syndrome | 40 | 0.4 | 1 | 39 |  |
| Gaucher disease | 66 | 0.7 | 39 | 27 |  |
| Idiopathie hypogonadotropic hypogonadism | 88 | 0.9 | 83 | 5 |  |
| Langerhans cell histiocytosis | 51 | 0.5 | 42 | 9 |  |
| Tetrahydrobioptein deficiency | 17 | 0.2 | 4 | 13 |  |
| Dravet syndrome | 9 | 0.1 | 0 | 9 |  |
| Niemann-Pick disease | 13 | 0.1 | 1 | 12 |  |
| Idiopathic pulmonary artery hypertension | 29 | 0.3 | 24 | 5 |  |
| Homozygote familial hypercholesterolemia | 20 | 0.2 | 10 | 10 |  |
|  |  |  |  |  |  |
|  | Mean | SD | Mean | Mean | p-value |
| Perceived severity of disease (score 1-10) | 8.1 | 2.3 | 7.9(2.3) | 8.6(2.0) | <.001 |
