# Supplementary material for: Measurement of Health-Related Quality of Life in Individuals With Rare Diseases in China: Nation-Wide Online Survey
Source: JMIR Public Health Surveill. 2023 Oct 31;9:e50147. doi: 10.2196/50147 (PMC10646671; doi:10.2196/50147)
Supplement: Multimedia Appendix 3 [file publichealth_v9i1e50147_app3.docx]

**Multimedia Appendix 3.** EQ-5D-5L utility scores reported by specific rare diseases.

| **Types of RDs** | |  |  |  |
| --- | --- | --- | --- | --- |
| **Overall sample** | |  |  |  |
|  | Amyotrophic lateral sclerosis | 0.154 (0.387) | N/A | N/A |
|  | Duchenne muscular dystrophy | 0.195 (0.335) | 0.041 (−0.013 to 0.095) | .14 |
|  | Spinal muscular atrophy | 0.230 (0.353) | 0.076 (0.020 to 0.133) | .008 |
|  | Mucopolysaccharidosis type I | 0.339 (0.369) | 0.185 (0.107 to 0.264) | <.001 |
|  | Huntington’s disease | 0.381 (0.426) | 0.228 (0.175 to 0.280) | <.001 |
|  | Pompe disease | 0.384 (0.368) | 0.230 (0.164 to 0.296) | <.001 |
|  | Spinocerebellar ataxia | 0.439 (0.354) | 0.285 (0.229 to 0.342) | <.001 |
|  | Niemann-Pick disease | 0.519 (0.465) | 0.365 (0.207 to 0.523) | <.001 |
|  | Spinal and bulbar muscular atrophy | 0.545 (0.281) | 0.391 (0.336 to 0.446) | <.001 |
|  | Hemophilia | 0.568 (0.319) | 0.414 (0.372 to 0.456) | <.001 |
|  | Osteogenesis imperfecta | 0.624 (0.303) | 0.470 (0.405 to 0.536) | <.001 |
|  | Epidermolysis bullosa | 0.624 (0.323) | 0.471 (0.417 to 0.524) | <.001 |
|  | Neuromyelitis optica spectrum disorders | 0.646 (0.323) | 0.492 (0.442 to 0.542) | <.001 |
|  | Gaucher disease | 0.673 (0.273) | 0.520 (0.441 to 0.598) | <.001 |
|  | Idiopathic pulmonary artery hypertension | 0.680 (0.216) | 0.526 (0.416 to 0.636) | <.001 |
|  | Lymphangioleiomyomatosis | 0.681 (0.280) | 0.527 (0.474 to 0.579) | <.001 |
|  | Fabry disease | 0.682 (0.227) | 0.529 (0.472 to 0.585) | <.001 |
|  | Dravet syndrome | 0.707 (0.188) | 0.553 (0.365 to 0.742) | <.001 |
|  | Multiple sclerosis | 0.723 (0.266) | 0.569 (0.525 to 0.614) | <.001 |
|  | Myasthenia gravis | 0.725 (0.271) | 0.571 (0.530 to 0.612) | <.001 |
|  | Homozygote familial hypercholesterolemia | 0.740 (0.227) | 0.586 (0.456 to 0.715) | <.001 |
|  | Hepatolenticular degeneration | 0.742 (0.299) | 0.588 (0.540 to 0.637) | <.001 |
|  | Scleroderma | 0.749 (0.224) | 0.596 (0.552 to 0.639) | <.001 |
|  | Prader-Willi syndrome | 0.767 (0.184) | 0.613 (0.518 to 0.709) | <.001 |
|  | Tuberous sclerosis complex | 0.768 (0.245) | 0.614 (0.562 to 0.667) | <.001 |
|  | Marfan syndrome | 0.771 (0.226) | 0.617 (0.573 to 0.660) | <.001 |
|  | Langerhans cell histiocytosis | 0.815 (0.222) | 0.661 (0.574 to 0.748) | <.001 |
|  | Tetrahydrobiopterin deficiency | 0.836 (0.118) | 0.683 (0.543 to 0.822) | <.001 |
|  | Idiopathic hypogonadotropic hypogonadism | 0.855 (0.116) | 0.701 (0.630 to 0.772) | <.001 |
|  | Albinism | 0.859 (0.158) | 0.705 (0.644 to 0.766) | <.001 |
|  | Phenylketonuria | 0.868 (0.215) | 0.714 (0.663 to 0.766) | <.001 |
|  | Kallmann syndrome | 0.876 (0.108) | 0.722 (0.662 to 0.783) | <.001 |
|  | Congenital adrenal hyperplasia | 0.954 (0.065) | 0.800 (0.723 to 0.877) | <.001 |
| **Self-completed sample** | | |  |  |
|  | Spinal muscular atrophy | 0.299 (0.363) | N/A | N/A |
|  | Duchenne muscular dystrophy | 0.322 (0.368) | 0.023 (−0.119 to 0.165) | .75 |
|  | Amyotrophic lateral sclerosis | 0.330 (0.352) | 0.031 (−0.051 to 0.113) | .45 |
|  | Pompe disease | 0.344 (0.364) | 0.045 (−0.033 to 0.122) | .26 |
|  | Spinocerebellar ataxia | 0.517 (0.301) | 0.218 (0.149 to 0.287) | <.001 |
|  | Hemophilia | 0.532 (0.316) | 0.234 (0.177 to 0.290) | <.001 |
|  | Mucopolysaccharidosis type I | 0.544 (0.271) | 0.245 (0.094 to 0.396) | <.001 |
|  | Spinal and bulbar muscular atrophy | 0.563 (0.252) | 0.265 (0.197 to 0.332) | <.001 |
|  | Osteogenesis imperfecta | 0.650 (0.285) | 0.351 (0.274 to 0.429) | <.001 |
|  | Neuromyelitis optica spectrum disorders | 0.653 (0.314) | 0.354 (0.292 to 0.416) | <.001 |
|  | Idiopathic pulmonary artery hypertension | 0.657 (0.219) | 0.358 (0.241 to 0.475) | <.001 |
|  | Epidermolysis bullosa | 0.657 (0.310) | 0.359 (0.292 to 0.425) | <.001 |
|  | Lymphangioleiomyomatosis | 0.678 (0.283) | 0.379 (0.317 to 0.442) | <.001 |
|  | Gaucher disease | 0.681 (0.277) | 0.382 (0.284 to 0.479) | <.001 |
|  | Huntington’s disease | 0.681 (0.399) | 0.382 (0.280 to 0.483) | <.001 |
|  | Homozygote familial hypercholesterolemia | 0.689 (0.227) | 0.390 (0.220 to 0.559) | <.001 |
|  | Fabry disease | 0.695 (0.198) | 0.396 (0.327 to 0.465) | <.001 |
|  | Multiple sclerosis | 0.736 (0.248) | 0.437 (0.380 to 0.495) | <.001 |
|  | Myasthenia gravis | 0.738 (0.256) | 0.439 (0.384 to 0.494) | <.001 |
|  | Scleroderma | 0.747 (0.207) | 0.448 (0.391 to 0.505) | <.001 |
|  | Hepatolenticular degeneration | 0.772 (0.237) | 0.474 (0.411 to 0.536) | <.001 |
|  | Marfan syndrome | 0.782 (0.220) | 0.483 (0.425 to 0.541) | <.001 |
|  | Tuberous sclerosis complex | 0.826 (0.132) | 0.527 (0.454 to 0.600) | <.001 |
|  | Langerhans cell histiocytosis | 0.833 (0.161) | 0.534 (0.439 to 0.630) | <.001 |
|  | Idiopathic hypogonadotropic hypogonadism | 0.857 (0.114) | 0.558 (0.481 to 0.636) | <.001 |
|  | Albinism | 0.860 (0.157) | 0.561 (0.490 to 0.632) | <.001 |
|  | Kallmann syndrome | 0.879 (0.098) | 0.580 (0.510 to 0.650) | <.001 |
|  | Tetrahydrobiopterin deficiency | 0.883 (0.074) | 0.584 (0.324 to 0.844) | <.001 |
|  | Phenylketonuria | 0.903 (0.178) | 0.604 (0.503 to 0.704) | <.001 |
|  | Congenital adrenal hyperplasia | 0.920 (0.102) | 0.621 (0.483 to 0.759) | <.001 |
| **Proxy-completed sample** | |  |  |  |
|  | Amyotrophic lateral sclerosis | 0.060 (0.373) | N/A | N/A |
|  | Spinal muscular atrophy | 0.166 (0.333) | 0.106 (0.023 to 0.189) | .01 |
|  | Duchenne muscular dystrophy | 0.186 (0.332) | 0.126 (0.057 to 0.195) | <.001 |
|  | Spinocerebellar ataxia | 0.212 (0.399) | 0.152 (0.046 to 0.258) | .005 |
|  | Mucopolysaccharidosis type I | 0.289 (0.374) | 0.229 (0.128 to 0.330) | <.001 |
|  | Huntington’s disease | 0.336 (0.412) | 0.275 (0.207 to 0.344) | <.001 |
|  | Spinal and bulbar muscular atrophy | 0.491 (0.350) | 0.431 (0.332 to 0.531) | <.001 |
|  | Pompe disease | 0.503 (0.357) | 0.443 (0.314 to 0.572) | <.001 |
|  | Niemann-Pick disease | 0.508 (0.484) | 0.448 (0.262 to 0.635) | <.001 |
|  | Epidermolysis bullosa | 0.529 (0.343) | 0.469 (0.372 to 0.566) | <.001 |
|  | Osteogenesis imperfecta | 0.550 (0.343) | 0.490 (0.363 to 0.617) | <.001 |
|  | Neuromyelitis optica spectrum disorders | 0.619 (0.361) | 0.559 (0.464 to 0.654) | <.001 |
|  | Fabry disease | 0.643 (0.298) | 0.583 (0.475 to 0.691) | <.001 |
|  | Multiple sclerosis | 0.654 (0.338) | 0.594 (0.515 to 0.673) | <.001 |
|  | Hemophilia | 0.656 (0.312) | 0.596 (0.533 to 0.660) | <.001 |
|  | Gaucher disease | 0.663 (0.273) | 0.603 (0.472 to 0.734) | <.001 |
|  | Myasthenia gravis | 0.674 (0.318) | 0.614 (0.552 to 0.676) | <.001 |
|  | Hepatolenticular degeneration | 0.676 (0.396) | 0.616 (0.537 to 0.695) | <.001 |
|  | Dravet syndrome | 0.707 (0.188) | 0.647 (0.434 to 0.860) | <.001 |
|  | Tuberous sclerosis complex | 0.728 (0.294) | 0.667 (0.593 to 0.742) | <.001 |
|  | Langerhans cell histiocytosis | 0.728 (0.406) | 0.668 (0.455 to 0.881) | <.001 |
|  | Marfan syndrome | 0.750 (0.235) | 0.689 (0.624 to 0.754) | <.001 |
|  | Scleroderma | 0.758 (0.276) | 0.698 (0.628 to 0.768) | <.001 |
|  | Lymphangioleiomyomatosis | 0.762 (0.142) | 0.702 (0.462 to 0.941) | <.001 |
|  | Prader-Willi syndrome | 0.778 (0.174) | 0.717 (0.605 to 0.830) | <.001 |
|  | Homozygote familial hypercholesterolemia | 0.791 (0.227) | 0.730 (0.528 to 0.933) | <.001 |
|  | Idiopathic pulmonary artery hypertension | 0.792 (0.174) | 0.732 (0.450 to 1.013) | <.001 |
|  | Idiopathic hypogonadotropic hypogonadism | 0.819 (0.164) | 0.759 (0.478 to 1.040) | <.001 |
|  | Tetrahydrobiopterin deficiency | 0.822 (0.127) | 0.762 (0.582 to 0.942) | <.001 |
|  | Kallmann syndrome | 0.847 (0.184) | 0.787 (0.608 to 0.967) | <.001 |
|  | Albinism | 0.853 (0.169) | 0.793 (0.647 to 0.938) | <.001 |
|  | Phenylketonuria | 0.863 (0.220) | 0.803 (0.735 to 0.870) | <.001 |
|  | Congenital adrenal hyperplasia | 0.964 (0.045) | 0.904 (0.803 to 1.005) | <.001 |
